# Circulating healing (CH) cells expressing BST2 are functionally activated by the injury-regulated systemic factor HGFA

**DOI:** 10.1186/s13287-018-1056-1

**Published:** 2018-11-08

**Authors:** Claudia Lo Sicco, Daniele Reverberi, Federico Villa, Ulrich Pfeffer, Rodolfo Quarto, Ranieri Cancedda, Roberta Tasso

**Affiliations:** 1 Cellular Oncology Laboratory, Department of Experimental Medicine (DIMES), University of Genova, Largo Rosanna Benzi 10, 16132 Genova, Italy; 2 U.O. Molecular Pathology, IRCCS Ospedale Policlinico San Martino, Largo Rosanna Benzi 10, 16132 Genova, Italy; 3 U.O. Cellular Oncology, IRCCS Ospedale Policlinico San Martino, Largo Rosanna Benzi 10, 16132 Genova, Italy; 4 Biorigen srl, Largo Rosanna Benzi 10, 16132 Genova, Italy

**Keywords:** Endogenous regeneration, Hepatocyte growth factor-A, Injury-regulated stimuli, Progenitor cells

## Abstract

**Background:**

Restoration of damaged tissues through the activation of endogenous progenitors is an attractive therapeutic option. A deep evaluation of the intrinsic stem/progenitor cell properties as well as the reciprocal interactions with injured environments is of critical importance.

**Methods:**

Here, we show that bone marrow stromal cell antigen 2 (BST2) allows the isolation of a population of circulating progenitors, the circulating healing (CH) cells, characterized by a distinctive core signature. The bone marrow (BM) origin of BST2^pos^ CH cells has been strengthened by the co-expression of leptin receptor, the hallmark of a subpopulation of BM-skeletal stem cells.

**Results:**

BST2^pos^ CH cells retained the capacity to (i) respond to injury signals generated by a bone fracture, (ii) modify the expression of cell motility genes following damage, and (iii) react to hepatocyte growth factor-activator (HGFA), an injury-related stimulus sufficient to induce their transition into G_ALERT_, a state in which cells are functionally activated and participate in tissue repair.

**Conclusions:**

Taken together, these results could pave the way for the identification of new strategies to enhance and potentiate endogenous regenerative mechanisms for future therapies.

**Electronic supplementary material:**

The online version of this article (10.1186/s13287-018-1056-1) contains supplementary material, which is available to authorized users.

## Background

To date, clinical efforts towards tissue—and in particular skeletal tissue—regeneration have focused on cell-based therapies, including bone marrow- and adipose-derived mesenchymal stromal cells (MSCs), or mature osteoblasts [1]. Despite initial encouraging results have been obtained with the transplantation of exogenous progenitors in different experimental models, their application in the clinical setting is still hampered by skepticism, fueled by problems regarding safety, immunogenicity, and questionable engraftment/differentiation ability of transplanted cells [2]. Following injury, the specific activation and mobilization of proper endogenous progenitors could represent a significant furtherance in the field of regenerative medicine, becoming the gist of future therapeutic approaches. Therefore, in-depth functional characterization of the activated and mobilized cells is a matter of critical relevance in order to exploit them and strengthen the body’s own regenerative potential [3]. Recent studies clearly indicate that discrete populations of osteogenic progenitor cells exist, with distinctive identities and specific differentiation abilities, suggesting both an alternative hierarchical model and the existence of multiple tissue-specific progenitors [4].

It is well known that a coordinated sequence of interactions between the environments in which progenitor cells reside in a quiescent state, namely specialized niches, and the injury-associated signals can instruct the cells migrating toward a damaged tissue [5, 6]. This is mainly due to the peculiar ability of progenitor cells to pick up extrinsic signals released by the niche as well as the injury, critically affecting their function [7, 8]. Undeniably, the activation of quiescent stem cells into the cell cycle is a key step in initiating the process of tissue repair [9, 10]. In this context, it has been recently shown that a population of circulating progenitors, defined as circulating healing (CH) cells, characterized by a lineage-negative/CD45-negative (Lin^neg^CD45^neg^) profile, possess a strong chemotactic potential, being responsive to the signals released by an injured environment as the one generated by a bone fracture [11]. Indeed, these stimuli direct their migration, with subsequent engraftment and differentiation into specific cells belonging to the affected tissue [11]. Here, we further implemented the phenotypic characterization of Lin^neg^CD45^neg^ CH cells and identified BST2 (bone marrow stromal cell antigen 2, also called CD317), a lipid raft-associated integral membrane protein, as a valuable CH cell marker that allowed their identification and defined their bone marrow (BM) compartmental origin. BM-derived BST2^pos^Lin^neg^CD45^neg^ cells co-expressed the leptin-receptor (LepR) cell surface antigen, hallmark of a subpopulation of quiescent adult bone-forming MSCs [12]. BST2-expressing CH cells maintained the capacity to (i) respond to injury environmental cues, actively proliferating and migrating toward the damaged site; (ii) modify the expression profile of specific cell motility-associated genes following the injury occurrence; and (iii) respond to the systemic factor hepatocyte growth factor activator (HGFA), a stimulus sufficient to induce the transition of multiple pools of quiescent progenitor cells into a G_ALERT_ state [13].

## Methods

### Mice

C57Bl/6 mice were purchased from Charles River Laboratories (Calco, Italy). Red fluorescent protein-transgenic (RFP-Tg) mice (B6.Cg-Tg(CAG-DsRed*MST) 1Nagy/J) were purchased from the Jackson Laboratory (Bar Harbor, MA, USA). We used female and male mice between 6 and 8 weeks of age. Chimeric mice were generated using lethally irradiated C57Bl/6 mice that were hematopoietically reconstituted with red fluorescent protein-positive (RFP^pos^) syngeneic bone marrow nucleated cells. Mice were bred and maintained at the Animal Facility of “IRCCS Ospedale Policlinico San Martino.” All animal procedures were approved by the “ IRCCS Ospedale Policlinico San Martino” Ethical Committee and performed in accordance with the national current regulations regarding the protection of animals used for scientific purpose (D. Lgs. 4 March 2014, n. 26, legislative transposition of Directive 2010/63/EU of the European Parliament and of the Council of 22 September 2010 on the protection of animals used for scientific purposes).

### Isolation of BST2^pos^ CH cells

CH cells were isolated from the peripheral blood (PB) and bone marrow (BM) of not-fractured (naive) wild-type (WT) and RFP-Tg mice, fractured mice at different times post-fracture induction (24, 36 h), and mice intravenously injected with recombinant hepatocyte growth factor activator (rHGFA) (24 and 36 h post-injection). Mice were anesthetized with an intraperitoneal (i.p.) injection of 100 μl/20 g body weight of stock solution containing ketamine HCl (100 mg/kg) and xylazine (10 mg/kg). PB was harvested from the retro-orbital vein and collected into heparin-coated tubes. BM cells were collected by flushing nucleated cells out of the femurs and tibiae with cold phosphate-buffered saline (PBS). Whole PB samples were lysed twice using a BD Pharm Lyse (BD Biosciences, Milan, Italy). Whole BM samples were lysed once using the same BD Pharm Lyse. Peripheral blood mononuclear cells (PBMCs) derived from each experimental group were pooled together (at least three mice/group) and stained for fluorescence-activated cell sorting (FACS) analysis. Similarly, BM-derived nucleated cells derived from each experimental group were pooled together (at least three mice/group) and stained for FACS analysis. Experiments were repeated at least six times.

### FACS and cell sorting

To determine the amount of BST2^pos^Lin^neg^CD45^neg^ cells, PBMCs and BM cells derived from fractured and naive mice were collected in FACS buffer (PBS containing 2% heat-inactivated fetal bovine serum) and stained for 10 min at 4 °C with PerCP-Cy5.5-conjugated Lineage Antibody Cocktail (BD Biosciences). After washing, cells were stained with APC rat anti-mouse CD45 antibody (clone 30-F11) (BD Biosciences) and with PE-Cy7 rat anti-mouse BST2 antibody (clone eBio927) (eBioscience, San Diego, CA, USA). BM-derived BST2^pos^Lin^neg^CD45^neg^ cells were also stained with the mouse Leptin R biotinylated antibody (Accession # Q3US58) (R&D Systems, Minneapolis, MN, Toll Free USA, Canada). A streptavidin Alexa Fluor 488 (Molecular Probes) was used for indirect staining to detect the biotinylated antibody. The percentage of BrdU^pos^ cells was analyzed using the PE anti-BrdU antibody (clone BU20A) (eBioscience) in combination with the FoxP3/Transcription Staining Buffer Set (eBioscience).

Fluorescence Minus One (FMO) controls were performed for each combination in order to set the best threshold in (sub)gating of BST2^pos^, LepR^pos^, and BrdU^pos^ subsets. A set of microsphere suspensions (2, 4, 6 μm) (Molecular Probes, Milan, Italy) was used as size references. BST2^pos^ CH cells were sorted with high purity mask, from naive and RFP-Tg mice. All experiments were performed on BD FACSAria II. Data were analyzed using BD FACSDiva software.

### Mouse femoral fracture model and BST2^pos^ CH cell injection

All surgical procedures were performed under anesthesia and normal sterile conditions. The injury model was performed as previously described [11]. The mice received post-operative analgesia (Buprenorphine 0.1 mg/kg), and unprotected weight-bearing was allowed immediately post-operation. One hundred and fifty thousand RFP^pos^BST2^pos^ CH cells were intravenously (i.v.) injected in four syngeneic WT mice 24 h post-fracture induction and in three syngeneic WT mice 40 days post-fracture induction.

### Cell quantification analysis

Cells present within the hard callus or knee region of fractured and cell-injected mice were counted with the ImageJ cell counting plugin. Three sections per animal were quantified. Results were expressed as percentage of cells for field.

### HGFA and BrdU administration

Purified recombinant-active HGFA (R&D systems, #1200-SE) was administered via intravenous tail vein injection in naive C57Bl/6 mice (*N* = 4) at a dose of 1 μg diluted into 100 μl of sterile PBS (vehicle solution). Control injections were performed using 100 μl of vehicle solution.

For BrdU incorporation assays, naive, fractured, or HGFA-injected mice (at least three mice each group) were given an i.p. injection of BrdU Labeling Reagent (Invitrogen) (10 μl/g body weight) every 8 h for 36 h. The frequency of BrdU^pos^ cells was then analyzed by flow cytometry, as previously described.

### Serum isolation and HGFA concentration

Serum was isolated from blood samples collected from the retro-orbital vein of both naive (*n* = 4) and fractured mice (24 h post-lesion induction) (*n* = 4). Blood was left to clot for 1 h at room temperature. The clot was removed by centrifugation (2000×*g* for 10 min, 4 °C), the serum was isolated by removing the upper clear layer of the blood sample, and it was maintained at − 80 °C until use. Serum HGFA levels were measured using the Mouse Hepatocyte growth factor activator (HGFAC) ELISA kit (Cusabio Biotech). Each replicate measurement represents a biologic replicate serum sample.

### RNA extraction, PCR array, and qPCR analysis

Total RNA was extracted from sorted BST2^pos^ CH cells derived from the PB and BM collected from naive, fractured (24 h post-lesion), and rHGFA-injected (24 h post-injection) mice using the RNeasy® Micro Kit (Qiagen, Milano, Italy) according to the manufacturer’s instructions. Noteworthy, BST2^pos^ CH cells were isolated only from the bone marrow flushed from the fractured leg. For the reverse transcription (RT) reactions and cDNA synthesis, 0.5 μg of total RNA was used in the RT^2^ First Strand kit (Qiagen) following the manufacturer’s instructions. The simultaneous expression profile of 84 key genes involved in cell motility was evaluated using the RT^2^ Profiler PCR Array Mouse Cell Motility (Qiagen) analysis using the PE ABI PRISM 7700 sequence detection system (Perkin-Elmer, Waltham, MA) and RT^2^ Sybr Green Mastermix (Qiagen). To validate the expression profile of selected genes, we performed a qPCR analysis. Each gene was tested on samples derived from 10 mice, and three independent experiments were performed. Primer sequences were designed using the NCBI Primer-Blast tool (http://www.ncbi.nlm.nih.gov/tools/primer-blast/). Gene expression levels were normalized using GAPDH as endogenous control by applying the 2−∆∆Ct method. Primer sequences were as follows: ***Hgf*** (FW. GGGATTCGCAGTACCCTCAC; REV. TCGGATGTTTGGGTCAGTGG); ***Fgf2*** (FW. GAGAAGAGCGACCCACACG; REV. ACACACTTAGAAGCCAGCAGC); ***Igf1*** (FW. GAAGCGATGGGGAAAATCAGC; REV. CGCCAGGTAGAAGAGGTGTG); ***Met*** (FW. AGGACAAGACCACCGAGGAT; REV. CCTCTGCACCAAGGACAACA); ***Mmp9*** (FW. GCGTCATTCGCGTGGATAAG; REV. TGGAAACTCACACGCCAGAA); ***Dpp4*** (FW. CTGGTGTGGATTTCCAAGCAAT; REV. AGCTATGGAGAGCTATGCTGTG); ***Ezr*** (FW. GAGGTAGAAGAGTGGCAGCA; REV. CCTCCTGCACGTGGTAATTCA); ***uPar*** (FW. GACCTCTGCAGGACTACCGT; REV. CATGGAGCCCATGCGGTAAC).

### Analysis of microarray gene expression profiling data

We used a previously generated dataset to identify genes associated with Lin^neg^CD45^neg^ CH cells isolated from the peripheral blood of naive mice (accession number: GSE64835). CH cell dataset was normalized to a collection of 33 publicly available microarray datasets corresponding to (i) three samples of an embryonic stem cell line (ESC l.) and three samples of very small embryonic-like (VSEL) stem cells (accession number: GSE29281), (ii) 12 samples of hematopoietic stem cells (HSC) at different stages of differentiation derived from GSE27787 and GSE47935 datasets, (iii) six samples of hemangioblasts (HEM) derived from dataset GSE43042, and (iv) three ESC primary culture (ESC) samples, three samples of multipotent adult progenitor cells (MAPC), and three samples of bone marrow-derived mesenchymal stromal cells (MSC) (accession number: GSE6933). Normalization of CH cell dataset with the abovementioned datasets was performed using RMA algorithms with quantile normalization implemented in R/BioConductor. Statistically significant expression changes between CH cells and selected comparison populations were based on pairwise comparisons and were determined using Significance Analysis of Microarrays (SAM) implemented in TMEV. For each pairwise comparison, genes regulated at least twofold were considered and the delta value was set to return a false discovery rate (FDR) of zero. After, those probes with fold change more than two, in any of the six pairwise comparisons, were selected to explore overlapping differentially upregulated genes among CH cells and the other selected populations by VENNTURE software (http://www.nia.nih.gov). The resulting 87 significantly upregulated genes were also visualized by hierarchical clustering using Pearson correlation and average linkage. Gene enrichment analysis was performed using EnrichR online tool.

### Immunofluorescence staining

To evaluate the migratory capacity through damaged tissues of PB-derived RFP^pos^BST2^pos^ CH cells, bilateral legs derived from fractured and cell-injected WT mice (*n* = 3), from fractured and PBS-injected WT mice (*n* = 3), were harvested 24 and 40 days post-lesion induction.

Bone samples were decalcified with a decalcifying solution (0.5 M EDTA, pH 7.5) for 24 h at 4 °C and cryoprotected with an ice-cold cryoprotection solution (20% sucrose, 2% polyvinylpyrrolidone) for 24 h at 4 °C. After washing with PBS, bone samples were embedded in OCT compound, snap frozen in liquid nitrogen, and stored at − 80 °C for immunofluorescence analysis. Sections of 5 μm were permeabilized with 0.3% Triton X-100 for 20 min and treated with a blocking buffer (1× PBS/5% normal goat serum/0.3% Triton X-100) for 60 min. Immunofluorescence staining was performed using a polyclonal anti-RFP antibody (Abcam) followed by goat anti-rabbit Alexa Fluor 633-conjugated secondary antibody (Thermo Scientific) and using a Runx2 rabbit mAb (Cell Signaling Technology, Milano, Italy) and a rabbit anti-mouse type II collagen polyclonal antibody (Millipore), both of them followed by a Alexa Fluor 594-conjugated goat anti-rabbit IgG secondary antibody (Thermo Scientific, Rockford, IL, USA). A DAPI solution was applied for 5 min for nuclear staining. Images were captured using an Axiovert 200 M microscope (Zeiss, Germany). Pre-immune controls have been performed.

### Statistical analysis

Assuming a Gaussian distribution of the data, we have used unpaired *t* test for the statistical analysis of differences between two groups and ordinary one-way ANOVA associated with Dunnett’s multiple comparisons test for analyzing differences among three or more groups. Statistical significance was set at *p* < 0.05. All statistical analyses were performed using GraphPad Prism Version 6.0a (GraphPad Software, La Jolla, CA, USA). The data analysis web portal at http://www.qiagen.com/geneglobe was applied to analyze PCR array datasets.

## Results

### Core signature of CH cells

**Fig. 1:**
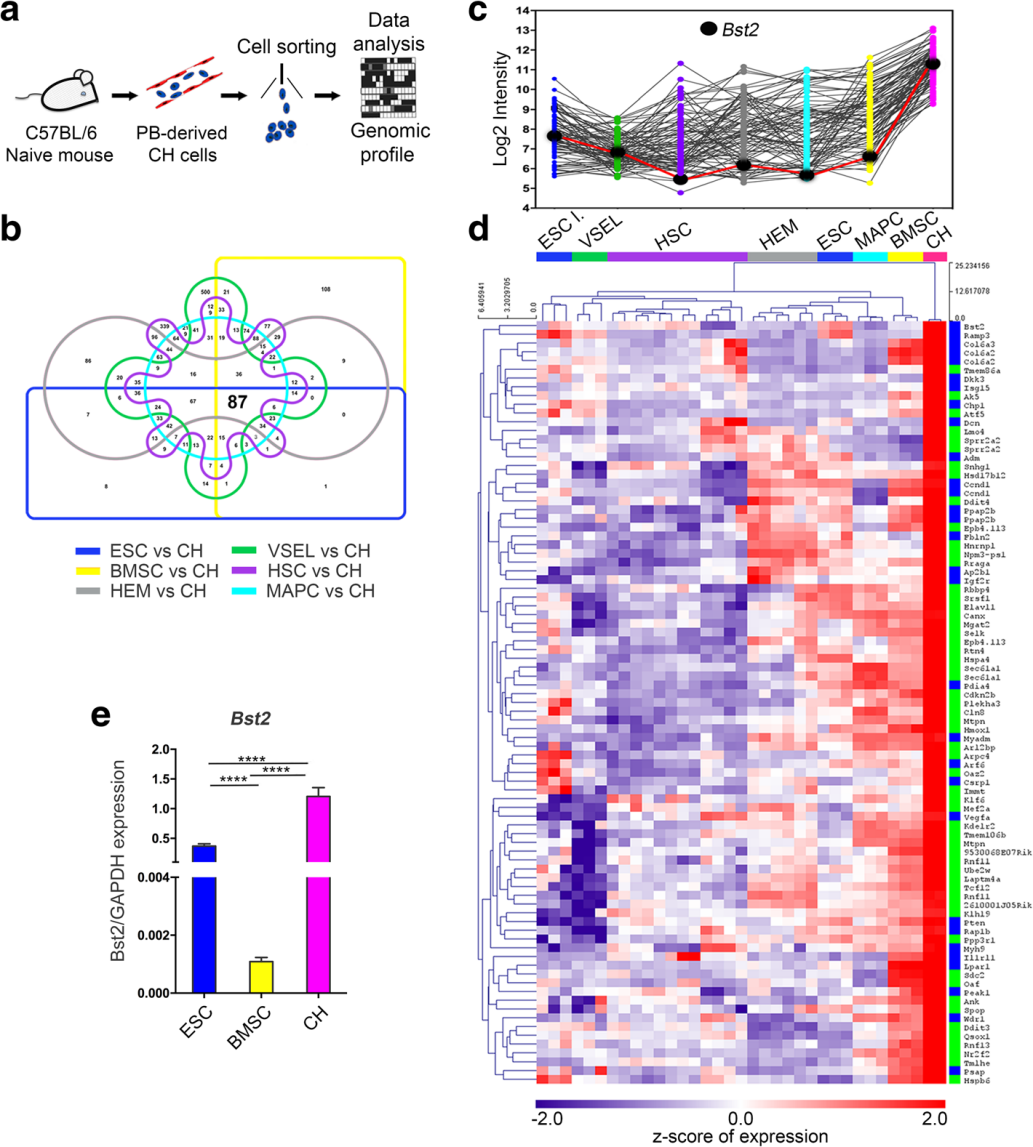
Transcriptome analysis reveals a core signature of CH cells. **a** Schematics illustrating isolation of CH cells from the PB of naive C57Bl/6 mice for transcriptome analysis. **b** Venn diagram identifying the common genes resulting from pairwise comparisons between CH cells (*n* = 2), used as reference group, and ESC (blue), (*n* = 6), BM-MSC (yellow) (*n* = 3), HEM (gray) (*n* = 6), VSEL (green) (*n* = 3), HSC (purple) (*n* = 12), and MAPC (light blue) (*n* = 3). **c** The histogram shows the log2 array intensity distribution of the 87 genes significantly upregulated in CH cells in comparison to the abovementioned cell populations. **d** Heat map showing the *z*-transformed expression values of the selected 87 genes together with their clustering into the “extracellular region” and “plasma membrane” gene ontology (GO) categories, indicated in blue. The genes not comprised in these two GO categories are indicated in green. **e** The significant expression of *Bst2* gene in CH cells was confirmed by quantitative PCR analysis, comparing the gene expression with BM-MSC and ESC (*t* value = 13.77, degree of freedom (df) = 7 and *t* value = 9.43, df = 7, respectively). ****p < 0.0001. Three independent replicates for each experimental group have been analyzed

We have previously reported the existence of a rare sub-population of circulating progenitors, Lin^neg^CD45^neg^ CH cells, actively participating in the fracture healing process and possessing a peculiar transcriptional expression pattern [11]. Despite the insights gained from this genome-wide analysis, the distinctive core signature of CH cells remained elusive. Here, we provide an integrative data analysis by comparing the already published transcriptome dataset of highly purified CH cells isolated from peripheral blood (PB) of naive mice (Fig. 1a) with publicly available profiles of other stem/progenitor cells characterized by different stemness degree and capabilities to enter the blood flow, including embryonic stem cells (ESC), very small embryonic-like stem cells (VSEL), hematopoietic stem cells (HSC), hemangioblasts (HEM), multipotent adult stem cells (MAPC), and bone marrow stromal cells (BMSC) [11]. To detect genes differentially expressed in CH cells, a Significance Analysis of Microarray (SAM) algorithm was applied for the six pairwise class comparisons, employing CH cell dataset as reference group (Additional file 1: Figure S1a–f). By means of VENNTURE [14], the analysis of the significantly upregulated genes (fold change > 2, FDR 0%) derived from each pairwise comparison allowed to single out a CH cell associated core signature composed of 87 characterizing genes (Fig. 1b). The actual intensity values on a log2 scale for each considered cell population underlies how these 87 genes are upregulated in CH cells with values ranging from 5.4 to 11.3 (Fig. 1c, Additional file 2: Table S1 and Additional file 3: Table S2). Unsupervised hierarchical clustering using Pearson correlation and average linkage revealed the distance of CH cells from the other populations, as identified by the length of the dendrogram branches (Fig. 1d and Additional file 4). Gene Ontology Enrichment Analysis (GOEA) identified, within the set of upregulated transcripts, 30 genes belonging to “extracellular region” and “plasma membrane” gene ontology categories (adherens junction, anchoring junction, cell surface, focal adhesion, cell-substrate adherens junction, cell-substrate junction, membrane coat, cell-cell junction). In particular, due to its recent identification as one of the key genes defining human bone marrow-mesenchymal stromal cell signature [15], we focused on *Bst2* (bone marrow stromal cell antigen 2), a gene encoding for a cell surface antigen whose expression was validated by quantitative real-time PCR (Fig. 1e).

### BST2 is an effective marker for CH cells

**Fig. 2:**
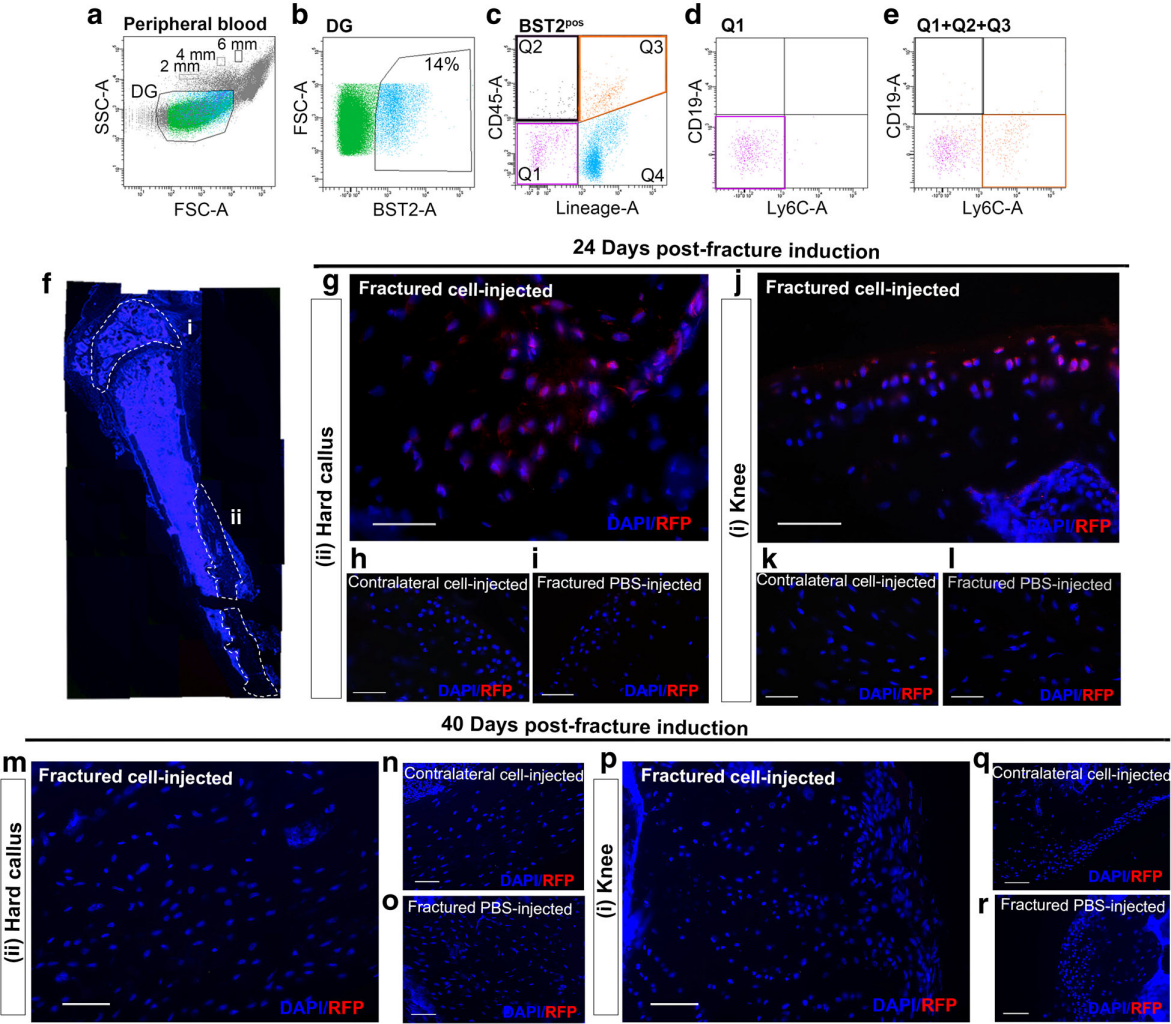
Small-sized BST2^pos^Lin^neg^CD45^neg^ cells circulate in the peripheral blood and engraft within injured bone and cartilage. **a–c** Representative flow cytometry strategy used to identify BST2^pos^Lin^neg^CD45^neg^ cells in the peripheral blood of C57Bl/6 mice (6 mice/experimental replicate; *n* = 15 independent experiments). DG, dimensional gate. BST2^pos^Lin^neg^CD45^neg^ cells (Q1) (**d**) as well as the sum of events falling within the BST2^pos^Lin^neg^CD45^neg^ cells (Q1), BST2^pos^Lin^neg^CD45^pos^ cells (Q2), and BST2^pos^Lin^pos^CD45^pos^ cells (Q3) (**e**) were stained with the B lymphocyte marker CD19 and the macrophage/dendritic cell precursor marker Ly6C. The peripheral blood of 30 naive C57Bl/6 mice (*n* = 5 independent experiments) has been considered. **f** Serial immunofluorescence sections (magnification × 5) from the resulting callus formed 24 days after the osteotomy. #knee, °° hard callus. **g–l** Double fluorescence images showing signals from DAPI (blue) and RFP (red) in the hard callus (**g**), in the contralateral femur (**h**), in the articular cartilage (**j**), and in the contralateral articular cartilage (**k**) of fractured mice that received RFP^pos^ CH cell injection 24 h post-fracture induction. Femur and articular cartilage of fractured and PBS-injected mice (**i** and **l**, respectively) were used as negative controls. Five mice/experimental group have been used. **m–r** Double fluorescence images showing signals from DAPI (blue) and RFP (red) in the hard callus (**m**), in the contralateral femur (**n**), in the articular cartilage (**p**), and in the contralateral articular cartilage (**q**) of fractured mice that received RFP^pos^ CH cell injection 40 days post-fracture induction. Femur and articular cartilage of fractured and PBS-injected mice (**o** and **r**, respectively) were used as negative controls. Three mice/experimental group have been used. Magnification × 40, scale bar 50 μm

As a step toward picking out BST2 as valuable cell surface antigen characterizing Lin^neg^CD45^neg^ CH cells, we used a flow cytometry approach similar to the one previously adopted to isolate these progenitors from the peripheral blood of adult mice [11]. Since CH cells represent a small-sized cell population with a diameter ranging from 2 to 6 μm [11], a specific dimensional gate (DG) has been delineated defining cutoff gates of 2, 4, and 6 μm using appropriate microsphere suspensions (Fig. 2a). An average of 14% events falling within the considered DG expressed BST2, as indicated in Fig. 2b (see also Additional file 1: Figure S2a, S2b). We then fractioned BST2-positive (BST2^pos^) events by differential expression of both CD45 and lineage markers, and we recognized the presence of BST2^pos^Lin^neg^CD45^neg^ cells resembling CH cells (Fig. 2c). However, BST2 antigen is also expressed by other two circulating sub-populations, the type I interferon (IFN)-producing cells (IPC), characterized by a Lin^pos^Ly-6C^pos^ phenotype and a subset of CD19^pos^CD45^pos^ plasma cells [16, 17]. To rule out the presence of contaminating IPC or plasma cells, we evaluated the co-expression of CD19 and Ly6C within the gate of interest (BST2^pos^Lin^neg^CD45^neg^). No BST2^pos^CD19^pos^ or BST2^pos^Ly6C^pos^ events were present in the Lin^neg^CD45^neg^ population (Fig. 2d). As expected, Ly6C^pos^ cells fell within the BST2^pos^Lin^pos^ events, whereas the few CD19^pos^ cells within the Lin^neg^CD45^pos^ events (Fig. 2e).

**Fig. 3:**
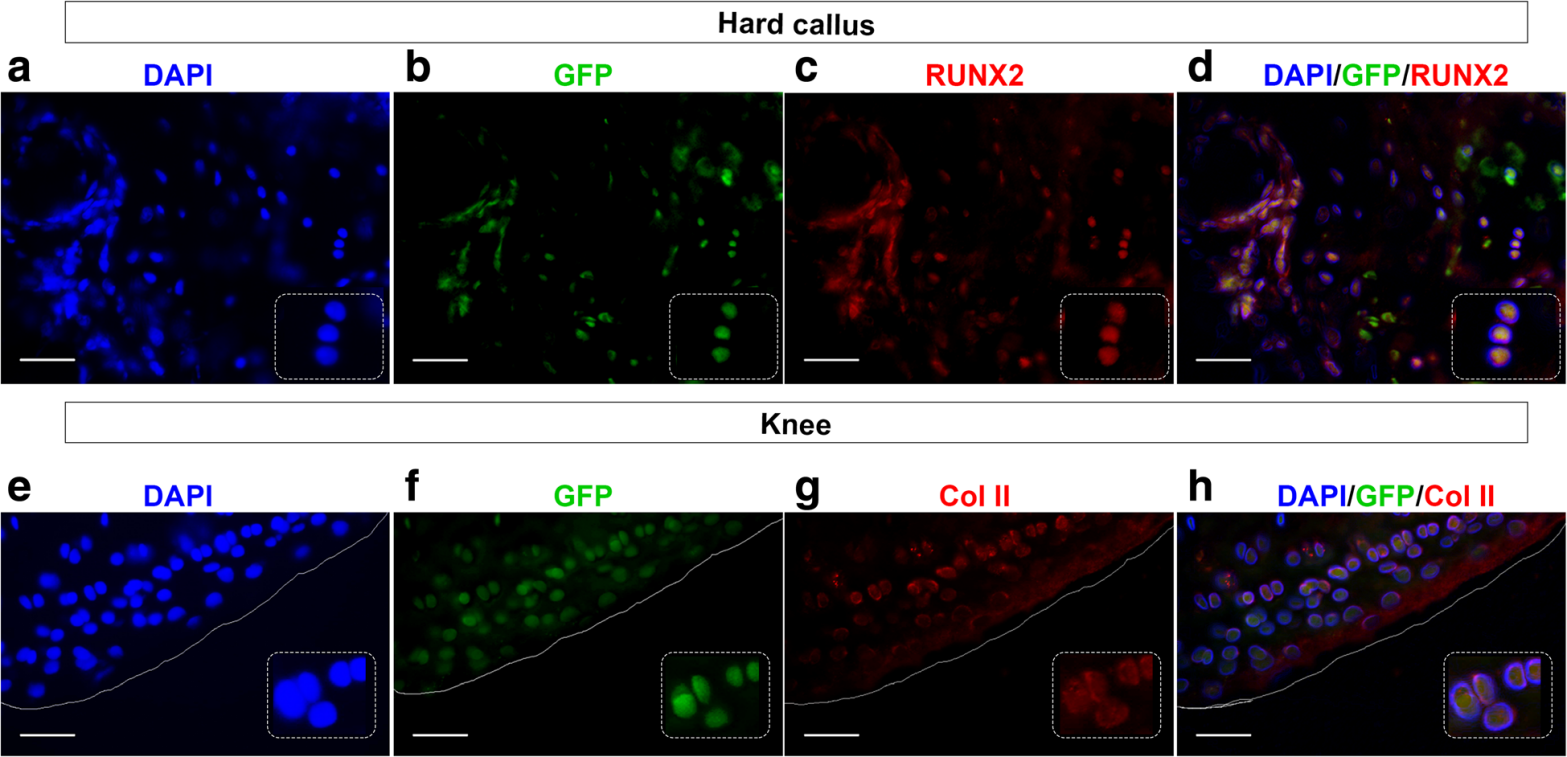
GFP^pos^ BST2^pos^-CH cells differentiate into tissue-specific cells. BST2^pos^-CH cells, isolated from the peripheral blood of GFP-transgenic naive mice and intravenously injected in syngeneic WT mice are able to engraft and differentiate in bone and cartilage tissue-specific cells, 24 days after. **a**–**d** Serial fluorescence images showing signals from DAPI (blue) (**a**), endogenous GFP (green) (**b**), and anti-Runx2 (red) (**c**) in the hard callus of fractured cell-injected mice. Overlap between DAPI, GFP, Runx2 is shown in panel **d**. White inset boxes in the panels show a higher magnification of representative cells co-expressing DAPI, GFP, and Runx2 signals. **e**–**h** Serial fluorescence images showing signals from DAPI (blue) (**e**), endogenous GFP (green) (**f**), and anti-Col II (red) (**g**) in knee region of fractured cell-injected mice. Overlap between DAPI, GFP, and Col II is shown in panel **h**. White inset boxes in the panels show a higher magnification of representative cells co-expressing DAPI, GFP, and Col II signals. Magnification × 40, scale bar 50 μm

To be de facto regarded as CH cells, other connotative functional characteristics have to be shared by PB-derived BST2^pos^Lin^neg^CD45^neg^ cells, that is, the capacity to be specifically mobilized toward an injured site and the ability to integrate into the specific tissue. We tested the cell homing capacity taking advantage of the same stabilized, transverse, mid-diaphyseal femoral fracture model previously adopted [11] (Fig. 2f). To trace their fate, BST2^pos^Lin^neg^CD45^neg^ cells have been sorted from red fluorescence protein (RFP)-transgenic mice using the same flow cytometry strategy described above (Additional file 1: Figure S2c–g), and RFP^pos^BST2^pos^Lin^neg^CD45^neg^ cells were intravenously (i.v.) injected in fractured mice 24 h post-lesion induction, during the acute phase response of the host to the injury event. After 24 days, when a hard callus was completely formed [18], we detected the presence of RFP^pos^ cells interspaced in the bone matrix belonging to the hard callus, as well as in the articular cartilage of fractured and cell-injected mice (Fig. 2g, j and Additional file 1: Figure S3a–h). Indeed, with regard to the presence of RFP^pos^ cells in the articular cartilage, it is to note that also this tissue was damaged during the surgical procedure, due to the insertion of the needle stabilizer. On the contrary, no RFP^pos^ cells have ever been detected either in the contralateral femurs of the same fractured and cell-injected mice or in the femurs of fractured and PBS-injected mice (Fig. 2h, i, k, l). The specificity of the anti-RFP antibody has been evaluated in sections of bone tissue derived from RFP-transgenic mice, as shown in Additional file 1: Figure. Moreover, injected BST^pos^-CH cells present in the hard callus of fractured mice co-expressed the early osteogenic transcription factor Runx2 (Fig. 3a–d and Additional file 1: Figure S3i–l), while BST^pos^-CH cells detected in the articular cartilage co-expressed type II collagen (Col II) (Fig. 3e–h and Additional file 1: Figure S3m–p), indicating their differentiation capability. When RFP^pos^BST2^pos^Lin^neg^CD45^neg^ cell transplantation was delayed to 40 days post-lesion induction, when an advanced remodeling phase has been reached and the molecular signals released by the injury event go to wane, transplanted cells did not ever migrate toward damaged tissues (Fig. 2m, p), clearly indicating that the timing of transplantation significantly affected the transplanted cell fate.

Taken together, these results reveal that (i) BST2 can be considered as a worthy identifying marker for CH cells, thus confirming the microarray data, and (ii) BST2^pos^ CH cells migrate to and engraft in wounded skeletal tissues only when transplanted during the acute phase response, in agreement with the concept that factor(s) released during this period are directly involved in progenitor cell activation.

### BST2^pos^ CH cells originate from the bone marrow and are specifically activated under injury conditions

**Fig. 4:**
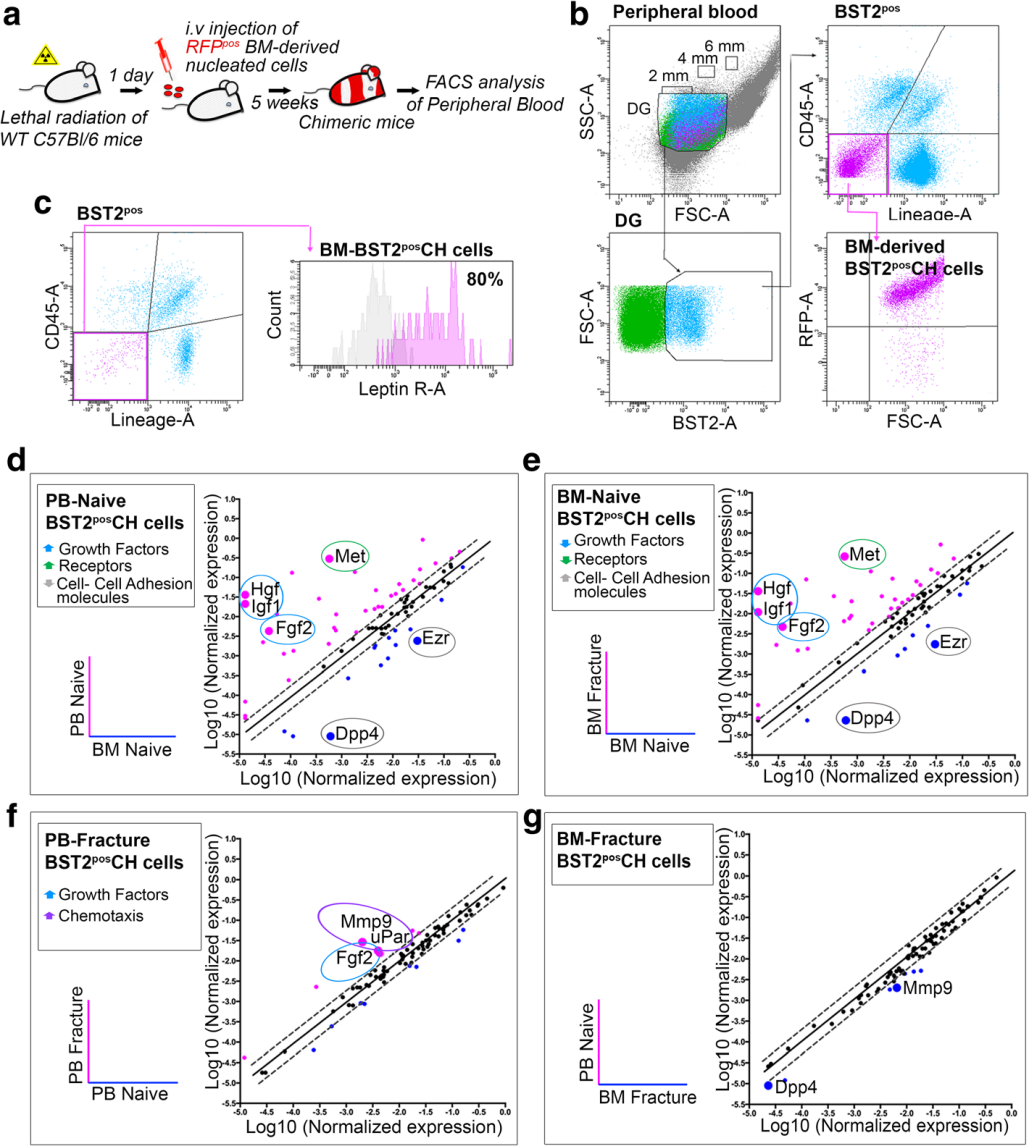
BST2^pos^ CH cells originate from bone marrow (BM) and modify their expression profile upon injury. **a** Schematic depicting the experimental procedures followed to analyze PB-derived cells in chimeric mice. **b** Representative flow cytometry analysis of BST2^pos^ CH cells recovered from the PB of chimeric mice (8 mice/experimental replicate; *n* = 3 independent experiments). **c** Leptin receptor (LepR) expression by BM-derived BST2^pos^Lin^neg^CD45^neg^ CH cells (6 mice/experimental replicate; *n* = 3 independent experiments). Area under pink line identifies cells reacting with specific antigen. Area under gray line indicates the Fluorescence Minus One (FMO) control. **d–g** Total RNA samples derived from BST2^pos^ CH cells isolated from the PB (*n* = 13) and BM (*n* = 10) of naive (PB-Naive and BM-Naive, respectively) and 24 h fractured (PB-Fracture (*n* = 13) and BM-Fracture (*n* = 10), respectively) mice and qRT-PCR assays were performed using Cell Motility RT2 Profiler PCR Array (3 independent experimental replicates). Each scatter plot compares the Log10 normalized expression of every gene on the array between PB-Naive (test group) and BM-Naive (reference group) (**d**), BM-Fracture (test group) and BM-Naive (reference group) (**e**), PB-Fracture (test group) and PB-Naive (reference group) (**f**), and PB-Naive (test group) and BM-Fracture (reference group) (**g**). The dashed lines indicate the diagonal and twofold change between each comparison. For each pairwise comparison, black points indicate unchanged gene expressions, pink points indicate upregulated genes, and blue points indicate downregulated genes in test groups

The major source of adult progenitors circulating in the PB is bone marrow (BM) [19]. Transplantation of syngeneic RFP-expressing BM cells into lethally irradiated mice has been used to verify the compartmental origin of circulating BST2^pos^ CH cells (Fig. 4a). Using the same flow cytometry approach described in Fig. 2a, it has been shown that BST2^pos^ CH cells isolated from the PB of chimeric mice were RFP-positive (RFP^pos^), indicating a BM origin (Fig. 4b). To confirm this, BST2^pos^ CH cells were detected in the BM of the same naive mice (Fig. 4c, left panel). CH cells retain distinctive features which are (i) the BM origin, (ii) the specific expression of BST2, and (iii) the ability to be specifically mobilized toward injured skeletal tissues. We therefore checked for the expression of leptin receptor (LepR), a marker used for the prospective identification of a subpopulation of skeletal stem cells [12]. More than 80% of BM-derived BST2^pos^ CH cells are LepR-positive (LepR^pos^), clarifying the factual nature of CH cells as early progenitors contributing to the fracture repair process (Fig. 4c, right panel).

To gain further insight into what distinguishes BST2^pos^ CH cells derived from PB and BM, and what dictates their responsiveness to injury-related signals, we investigated the profiles of a focused panel of genes involved in cell motility and activation differentially expressed by four experimental groups that are BST2^pos^ CH cells isolated from (i) PB of naive, not-fractured mice (PB-Naive); (ii) PB of fractured mice, 24 h post-lesion induction (PB-Fracture); (iii) BM of naive, not-fractured mice (BM-Naive); and (iv) BM of fractured mice, 24 h post-lesion induction (BM-Fracture). Gene expression changes were evaluated by plotting the normalized expression of each considered gene in one condition against the other (Fig. 4d–g and Additional file 5: Table S3). In this way, 36 genes resulted significantly upregulated and 14 downregulated in BST2^pos^ CH cells derived from PB-Naive in comparison to BM-Naive (Fig. 4d). Among the upregulated ones, four genes encoding for fibroblast growth factor 2 (*Fgf2)*, hepatocyte growth factor (*Hgf*), insulin-like growth factor 1(*Igf1*), and the receptor *Met* caught our attention. FGF2, HGF, and IGF1 are pleiotropic growth factors controlling cell proliferation and migration [20–22]. MET, a receptor tyrosine kinase that is activated by the binding of HGF, is required for the specific activation of stem/progenitor cells into a primed G_Alert_ state in which they possess an enhanced potential to activate themselves and promote tissue repair [13]. On the contrary, 14 genes resulted over-expressed in cells derived from the BM compartment, among which the two cell adhesion molecules *Dpp4* and *Ezr*, suggesting the importance of adhesion interactions in the anchorage and retention of quiescent progenitors to the bone marrow environment (Fig. 4d). The comparison between BST2^pos^ CH cells derived from BM-Naive and BM-Fracture led to similar results, with 35 genes significantly over-expressed upon fracture, including the growth factors and receptor mentioned above, and 10 downregulated genes, comprising those encoding for cell-adhesion molecules (Fig. 4e). These results suggest that the injury event can trigger profound changes in the motility signature of CH cells. Almost identical profiles came out from the comparison of PB-Naive and BM-Fracture, with the exception of *Dpp4* and *Mmp9* that resulted significantly downregulated in PB-Naive samples (Fig. 4f).

When we compared cells isolated from PB-Naive and PB-Fracture, little variations in their gene expression profiles emerged (Fig. 4g). In particular, *Mmp9*, *Fgf2*, and *uPar* were significantly upregulated in PB-derived cells belonging to fractured mice. The membrane receptor uPar and the endopeptidase MMP9 have been described to play important roles in physiological processes such as wound healing [23, 24] and stem cell migration [25, 26], confirming once again that the damage event is associated with variations in the expression profiles of those genes that are involved in a rapid response to injury. Trends of selected genes in the four experimental groups are reported in Additional file 1: Figure S5a–d.

### Systemic HGFA is a priming factor for BST2^pos^ CH cell functional response

**Fig. 5:**
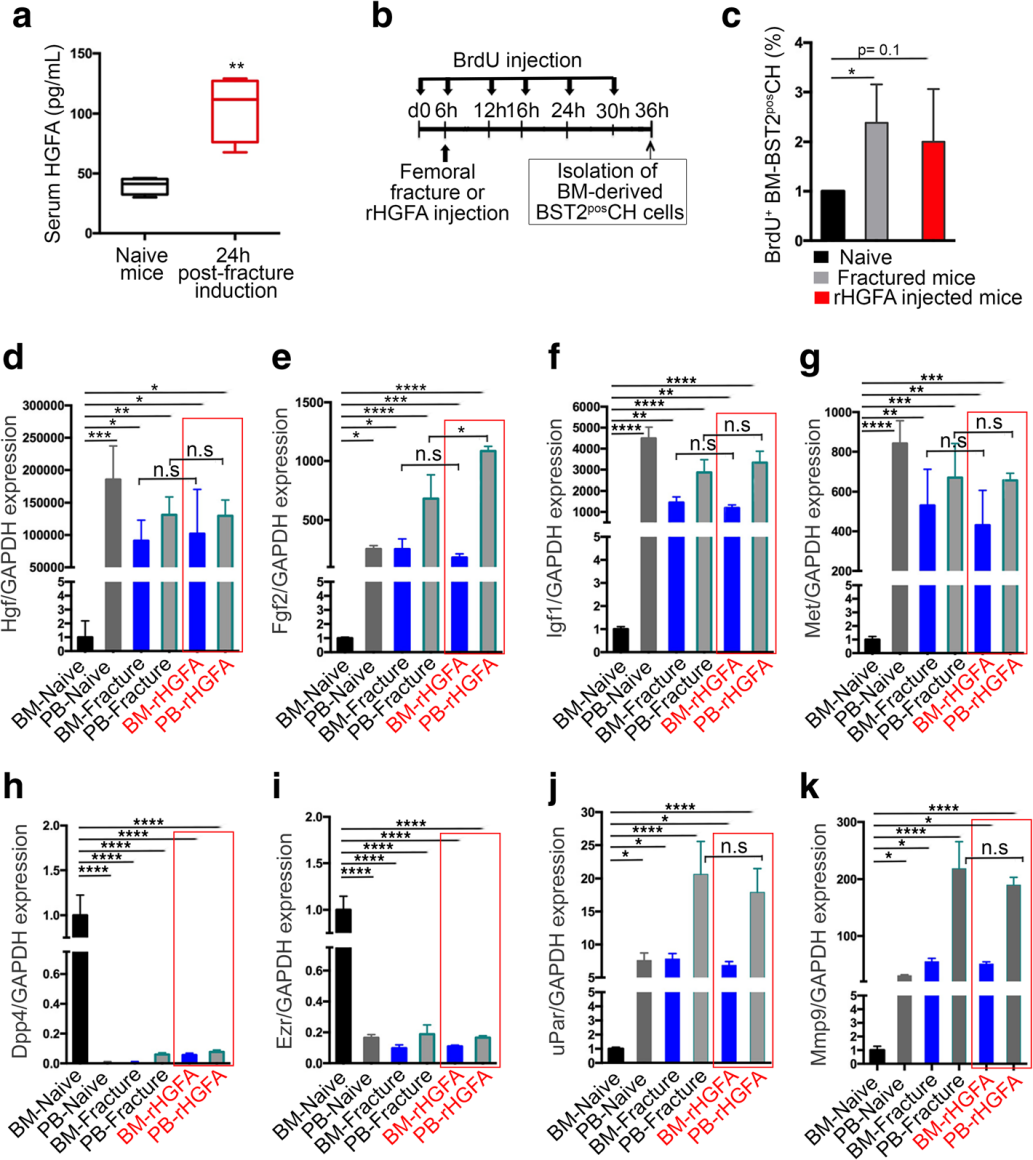
HGFA is sufficient to induce the G_ALERT_ transition and the functional molecular changes of BST2^pos^ CH cells. **a** Serum samples were prepared from non-injured (naive) or 24 h injured (fractured) mice, and HGFA levels were measured by ELISA. Bar graphs represent mean ± SD (*t* value = 4.62, df = 6). ** *p* = 0.0036. The serum from four mice/group have been analyzed. **b** Schematic depicting the timelines of BM-derived BST2^pos^ CH cell isolation from fractured or recombinant HGFA (rHGFA)-injected mice contemporary receiving consecutive BrdU injections. (**c**) BM-derived BST2^pos^ CH cells derived from fractured (gray bar) or rHGFA-injected (red bar) mice have higher propensity to cycle in vivo than cells extracted from naive control mice (black bar). Three mice/experimental group have been used and four independent replicates have been performed. Bar graphs represent mean ± SD (*t* = 3.11, df = 4) *p = 0.0357. **d–k** The expression of the selected motility-associated genes *Hgf* (**d**), *Fgf2* (**e**), *Igf1* (**f**), *Met* (**g**), *Dpp4* (**h**), *Ezr* (**i**), *uPar* (**j**), and *Mmp9* (**k**) was confirmed by quantitative PCR analysis, comparing the gene expression profiles of BST2^pos^ CH cells isolated from PB- and BM-Naive (14 mice), PB- and BM-Fracture (12 mice), and PB- and BM-rHGFA mice (six mice). Bar graphs represent mean ± SD, *p < 0.0306, **p < 0.0096, ****p < 0.0001

It has been recently reported that in response to injury-induced systemic signals, many stem/progenitor cells reversibly transition between two functional cellular phases, G_0_ and G_ALERT_ [13, 27]. During the G_ALERT_ state, cells possess an increased capacity to activate and participate in tissue repair processes. In particular, HGF-activator (HGFA), the primary HGF protease, is induced in response to injury and is one of the main factors responsible of this functional transition [13]. In naive mice, BST2^pos^ CH cells express significantly higher levels of the HGF-receptor tyrosine kinase *Met* when isolated from the PB than the BM (Fig. 4d). Similarly, *Met* expression is significantly higher in BST2^pos^ CH cells sorted from the BM of fractured mice than in the BM of naive mice (Fig. 4e). We tested whether the sera of injured mice contained higher levels of HGFA by ELISA. As reported in Fig. 5a, a significantly higher proportion of HGFA was measured in injured sera analyzed 24 h post-fracture induction in comparison to sera isolated from naive mice, suggesting a possible involvement of this systemic factor in CH cell activation in response to injury. To evaluate whether HGFA itself could mimic the injury-generated environment, potentially regulating the transition of BM-derived CH cells from a quiescent to an activated state, either a femoral fracture or a single administration of recombinant HGFA (rHGFA) was performed 6 h after BrdU (5-bromodeoxyuridine) injection in two parallel groups of animals. The BrdU injection was repeated every 6 h, for a total of 36 h (Fig. 5b). As measured by BrdU incorporation, BM-derived BST2^pos^ CH cells derived from fractured mice showed a significantly higher propensity to cycle if compared to naive control mice, and a similar trend was observed in cells derived from rHGFA-injected mice (Fig. 5c). To confirm the results obtained by PCR arrays, but also to assess the injury-mimicking effects of rHGFA, we compared gene expression profiles of the above-considered genes derived from PB and BM of naive, fractured, and rHGFA-injected mice (Fig. 5d–k). As expected, the expression profiles of *Hgf*, *Fgf2*, and *Igf1* were significantly upregulated in cells isolated from PB-Naive and from both PB- and BM-Fracture in comparison to the cells isolated from the BM of naive control mice (Fig. 5d–f). rHGFA administration resembled the outcomes prompted by bone fracture (Fig. 5d–f). Similarly, *Met* was over-expressed by BST2^pos^ CH cells extracted from PB-Naive, as well as from PB- and BM-Fracture (Fig. 5g). Again, rHGFA administration induced in both PB- and BM-derived cells a significant upregulation of this receptor, recapitulating the fracture-induced effect (Fig. 5g). As previously indicated by the PCR arrays, genes encoding for the cell-adhesion molecules Dpp4 and Ezr were significantly upregulated in cells extracted from BM-Naive in comparison to the other considered experimental groups, comprising the ones derived from rHGFA-injected mice (Fig. 5h, i). Finally, the genes encoding for the proteolytic molecules uPar and Mmp9 were over-expressed in PB-Fracture and PB-rHGFA-derived cells (Fig. 5j, k). Collectively, these data indicate that (i) the systemic factor HGFA induce the transition of CH cells into a G_ALERT_ state, similarly to its reported effect on other stem/progenitor cell populations, and (ii) injecting mice with rHGFA is sufficient to induce a series of molecular functional changes in BST2^pos^ CH cells, predisposing their functional response to injury.

## Discussion

Our data support the conclusion that CH cells are an endogenous source of progenitors characterized by a unique core signature, originating from the bone marrow compartment and expressing two characteristic cell-surface antigens, BST2 and LepR.

BST2 is an antiviral antigen expressed by different immune cell types that plays complex roles in the regulation of viral infection [28]. Recently, it has been reported as one of the 28 CD marker genes that define the molecular signature of human BM-derived MSCs [15], being involved in their osteogenic differentiation via the regulation of the BMP2 signaling pathway [29]. On the other hand, within the plethora of proposed markers, LepR represents an undeniable antigen that fulfills the minimal criteria for defining a subpopulation of skeletal stem cells [12]. LepR-expressing cells give rise to osteoblasts and adipocytes during fetal development and to chondrocytes only after a mechanical injury to articular cartilage or following tibial fracture in adult mice [4, 12].

To the best of our knowledge, we here report for the first time the existence of a rare population of BM-derived progenitors co-expressing LepR and BST2, the latter so far described only at mRNA level, traceable in the circulation of naive mice in response to normal physiological stimuli, and more importantly, activated under injury conditions. CH cells share the distinctive property of many adult progenitors to be retained in a quiescent, non-cycling state, until they are needed for maintaining tissue homeostasis or enhancing tissue repair [30]. As previously reported by Joseph T. Rodgers and colleagues, the stem cell quiescent state is composed of two distinct functional phases, G_0_ and G_ALERT_, among which the cells transition in response to injury-induced systemic signals [9]. In our experimental model, during the acute phase response of the host to damage, BM-derived CH cells start to proliferate and over-express specific genes related to motility pathways, leading, in the later phases of the healing process, to an efficient cell engraftment within the hard callus and the articular cartilage of injured mice. Among the injury-regulated systemic factors, the active form of HGF (HGFA) possesses the processing enzymatic activity of HGF and it is necessary and sufficient to induce the G_ALERT_ transition of several stem/progenitor cells [13].

The proof that injury induces the release of high levels of HGFA in the serum and that CH cells significantly over-express the gene encoding for the HGF-receptor Met when they circulate in physiologic conditions or when they are present in the bone marrow of injured mice has suggested that HGFA could be also involved in their priming. Indeed, the administration of a single dose of rHGFA mimicked the fracture effects, prompting the transitioning of CH cells to an alerted state and the functional changes implicated in the complex events underlying cell migration and activation. Nevertheless, other signaling pathways could be implicated in the activation of CH cells. Among the others, the Wnt/β-catenin pathway has been described to play a key role in the activation of tissue-resident stem cells, including human bone marrow-derived MSCs [31], as well as in the cell fate lineage specification in somatic stem cells [32]. Moreover, it has been reported that injury signals trigger the endogenous Wnt pathway, whose activation is typically rapid and spatially restricted to the site of damage [33].

Based on lineage tracing experiments, several stem/progenitor cell identities have been proposed, often with completely or partially overlapping characteristics [34, 35]. Only recently, advances for identifying human and mouse osteogenic progenitor cells have been reported [12, 36]. As a whole, the data here presented focus on the phenotypic and functional characterization of an endogenous cell population, without any intention of demonstrating a factual and active involvement in the repair process, in terms of enhancement of the repair itself. Many challenges have to be overcome before the regeneration or repair by endogenous progenitors can be succeeded. Multidisciplinary approaches aiming at regulating the proper migration, persistence, and differentiation of mobilized cells, without over-activating the progenitor cell pool, should be particularly important for developing novel and reliable self-repair strategies.

## Conclusions

Our collective data contribute to place CH cells as a particularly interesting source of endogenous progenitors with peculiar functional characteristics. The detailed understanding of both local and systemic signals able to skew the endogenous cell functional activities, perhaps rendering them more responsive to extrinsic cues, could open up new opportunities of advance in the translational regenerative medicine field.

## Additional files

Additional file 1: **Figure S1.** Pairwise class comparisons detecting genes differentially expressed in CH cells. **Figure S2.** Identification and sorting of BST2^pos^ CH cells. **Figure S3.** Quantification of BST2^pos^ CH cells migrated toward the injury sites. **Figure S4.** Specificity of the used anti-RFP antibody. **Figure S5.** Effects of injury-related signals on BST2^pos^ CH cells motility. (PDF 15587 kb)

Additional file 2: **Table S1.** Log2 Intensity values of the 87 genes characterizing the CH cells for each considered cell population. (XLSX 69 kb)

Additional file 3: **Table S2.** Fold change values of the 87 characterizing genes derived from SAM analysis for each pairwise class comparison. (XLSX 61 kb)

Additional file 4: Unsupervised hierarchical clustering of considered cell populations. (TXT 41 kb)

Additional file 5: **Table S3.** RT-PCR array data set for the four considered experimental groups. (XLSX 57 kb)

## Abbreviations

BM: Bone marrow
BMSC: Bone marrow stromal cells
BrdU: 5-bromodeoxyuridine
BST2: Bone marrow stromal cell antigen 2
CH: Circulating healing cells
Col II: Type II collagen
DG: Dimensional gate
DPP4: Dipeptidylpeptidase 4
ESC: Embryonic stem cells
EZR: Ezrin
FGF2: Fibroblast growth factor 2
GFP: Green fluorescence protein
GOEA: Gene Ontology Enrichment Analysis
HEM: Hemangioblasts
HGF: Hepatocyte growth factor
HGFA: Hepatocyte growth factor-activator
HSC: Hematopoietic stem cells
IFN: Interferon
IGF1: Insulin-like growth factor 1
LepR: Leptin-receptor
MAPC: Multipotent adult stem cells
MET: Met proto-oncogene
MMP9: Matrix metallopeptidase 9
MSC: Mesenchymal stromal cells
PB: Peripheral blood
RFP: Red fluorescence protein
rHGFA: Recombinant hepatocyte growth factor-activator
SAM: Significance analysis of microarray
uPAR: Plasminogen activator
VSEL: Very small embryonic-like stem cells
