# Supplementary material for: Circulating healing (CH) cells expressing BST2 are functionally activated by the injury-regulated systemic factor HGFA
Source: Stem Cell Res Ther. 2018 Nov 8;9:300. doi: 10.1186/s13287-018-1056-1 (PMC6225669; doi:10.1186/s13287-018-1056-1)
Supplement: Supplementary file 1 — Figure S1. Pairwise class comparisons detecting genes differentially expressed in CH cells. Figure S2. Identification and sorting of BST2pos CH cells. Figure S3. Quantification of BST2pos CH cells migrated toward the injury sites. Figure S4. Specificity of the used anti-RFP antibody. Figure S5. Effects of injury-related signals on BST2pos CH cells motility. (PDF 15587 kb) [file 13287_2018_1056_MOESM1_ESM.pdf]

Additional file 1

Circulating Healing (CH) cells expressing BST2 are functionally activated by the Injury-regulated systemic factor HGFA

Claudia Lo Sicco[1], Daniele Reverberi[2], Federico Villa[3], Ulrich Pfeffer[2], Rodolfo Quarto[1,3], Ranieri Cancedda[1,3] & Roberta Tasso[3 *]

[1] Laboratory of Regenerative Medicine, Department of Experimental Medicine (DIMES), University of Genova, Largo Rosanna Benzi 10, 16132 Genova, Italy; e-mail: claudia.losicco@gmail.com and rodolfo.quarto@unige.it

[2] U.O. Molecular Pathology, IRCCS Ospedale Policlinico San Martino, Largo Rosanna Benzi 10, 16132 Genova, Italy; e-mail: daniele.reverberi@hsanmartino.it and ulrich.pfeffer@hsanmartino.it

[3] Laboratory of Regenerative Medicine, IRCCS Ospedale Policlinico San Martino, Largo Rosanna Benzi 10, 16132 Genova, Italy; e-mail: vilfed@libero.it and robertatasso@gmail.com

[4] Biorigen srl, Largo Rosanna Benzi 10, 16132 Genova, Italy; e-mail: ranieri.cancedda@unige.it

Figure S1

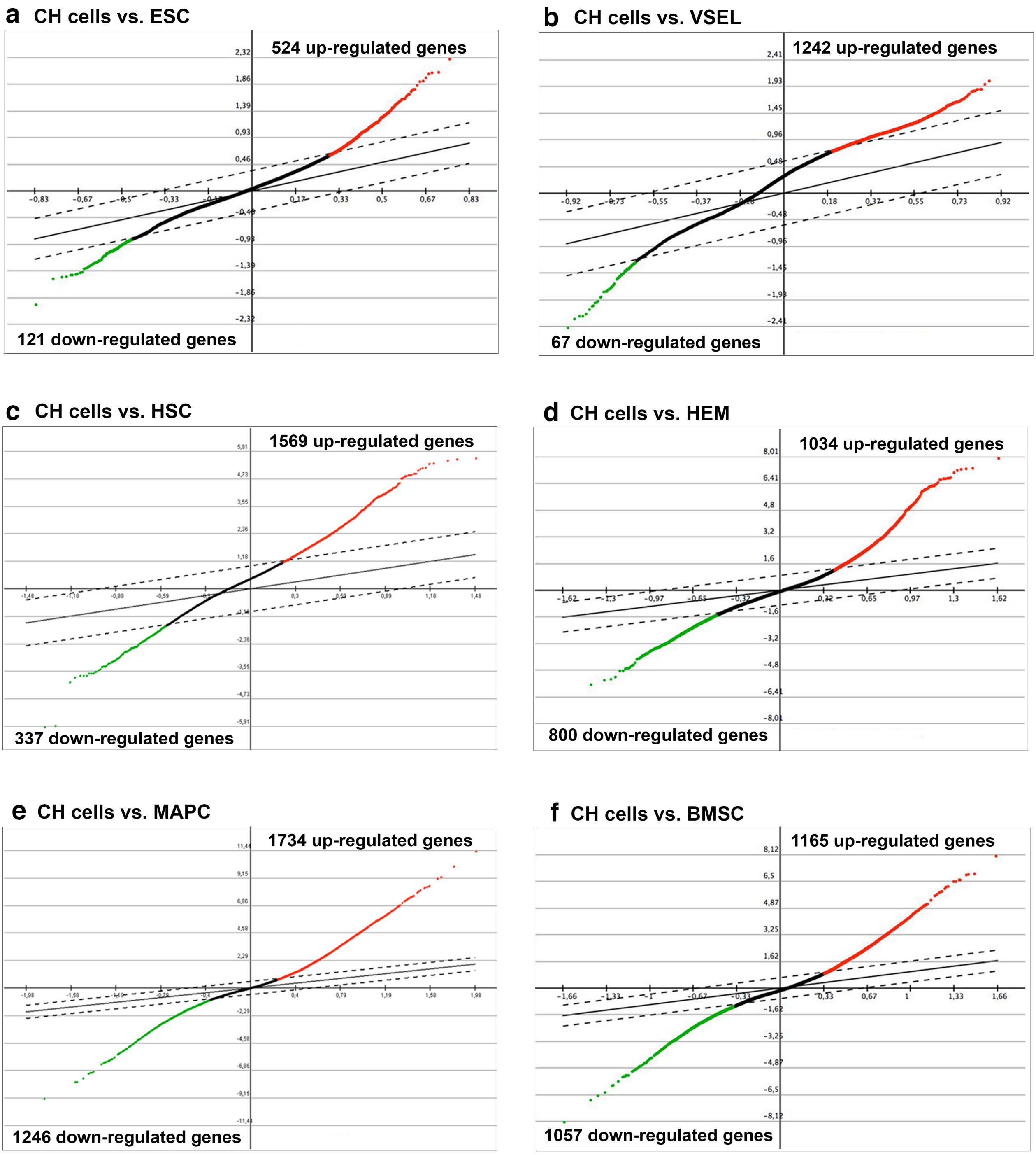

Figure S1. Pairwise class comparisons detecting genes differentially expressed in CH cells.

Scatter plots derives by Significance Analysis of Microarray (SAM) of pairwise comparison between CH cells, used as reference group, and Embryonic Stem Cells (ESC) (**a**), Very Small Embryonic-Like Stem Cells (VSEL) (**b**), Hematopoietic Stem Cells (HSC) (**c**), Hemangioblasts (HEM) (**d**), Multipotent Adult Stem Cells (MAPC) (**e**), and Bone Marrow Stromal Cells (BMSC)

(**f**). For each pairwise comparison, significant up-regulated and down-regulated genes in CH cells are indicated by red and green points, respectively. 2-fold threshold. False Discovery Rate 0%.

Figure S2

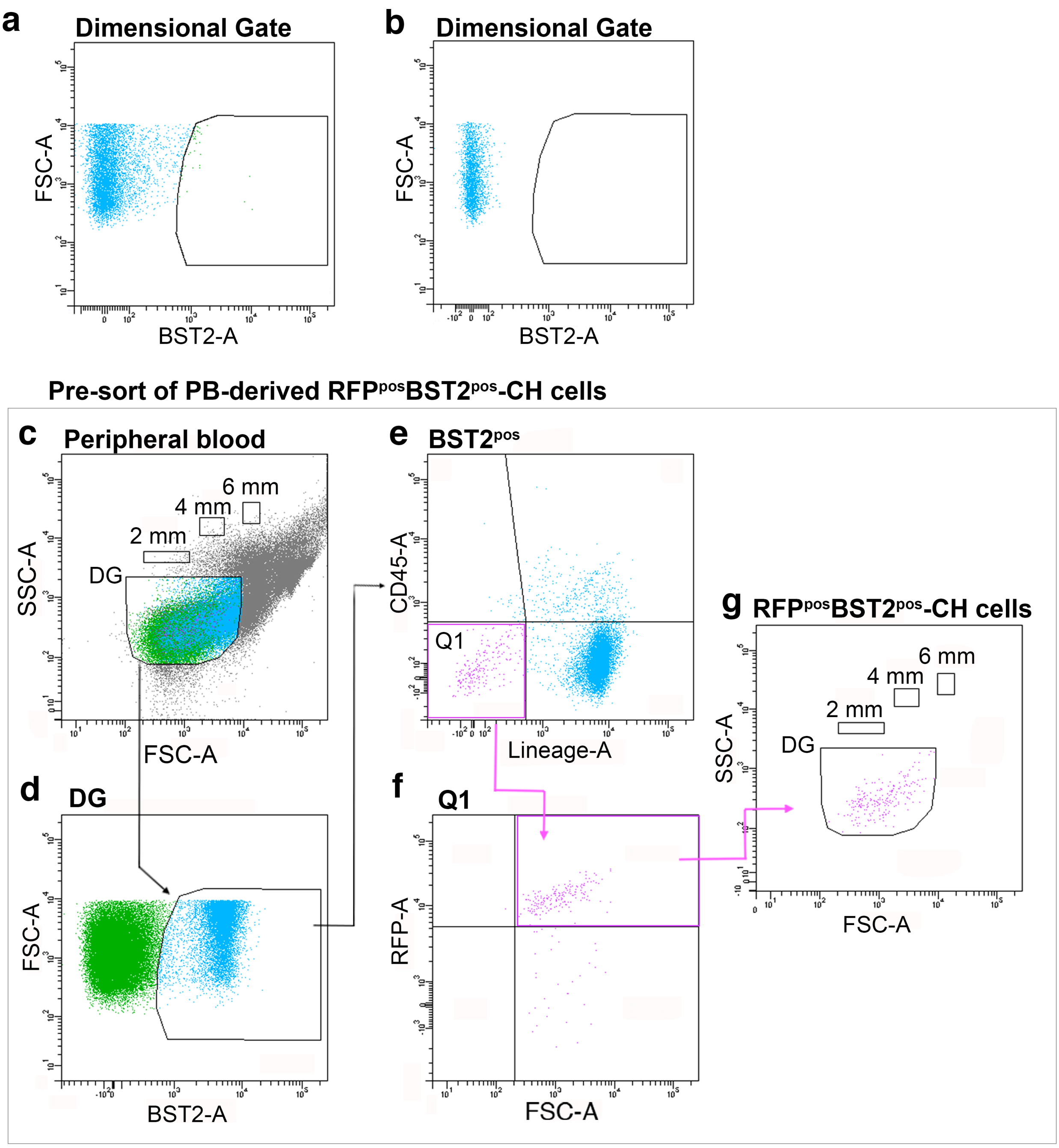

Figure S2. Identification and sorting of BST2^{pos} CH cells.

BST2 is a valuable antibody which allows CH cells enrichment. Representative flow cytometry dot plot shows Fluorescence Minus One (FMO) (**a**) and isotype control (**b**) of BST2 expression in

Peripheral blood (PB) cell suspension. Representative flow cytometry strategy used to sort BST2^{pos} CH cells from the peripheral blood of C57Bl/6 transgenic for the ubiquitous RFP expression. DG, dimensional gate (**c-f**). RFP^{pos}BST2^{pos}-CH cells are characterized by small-sized and fall exactly in the selected DG (**g**).

Figure S3

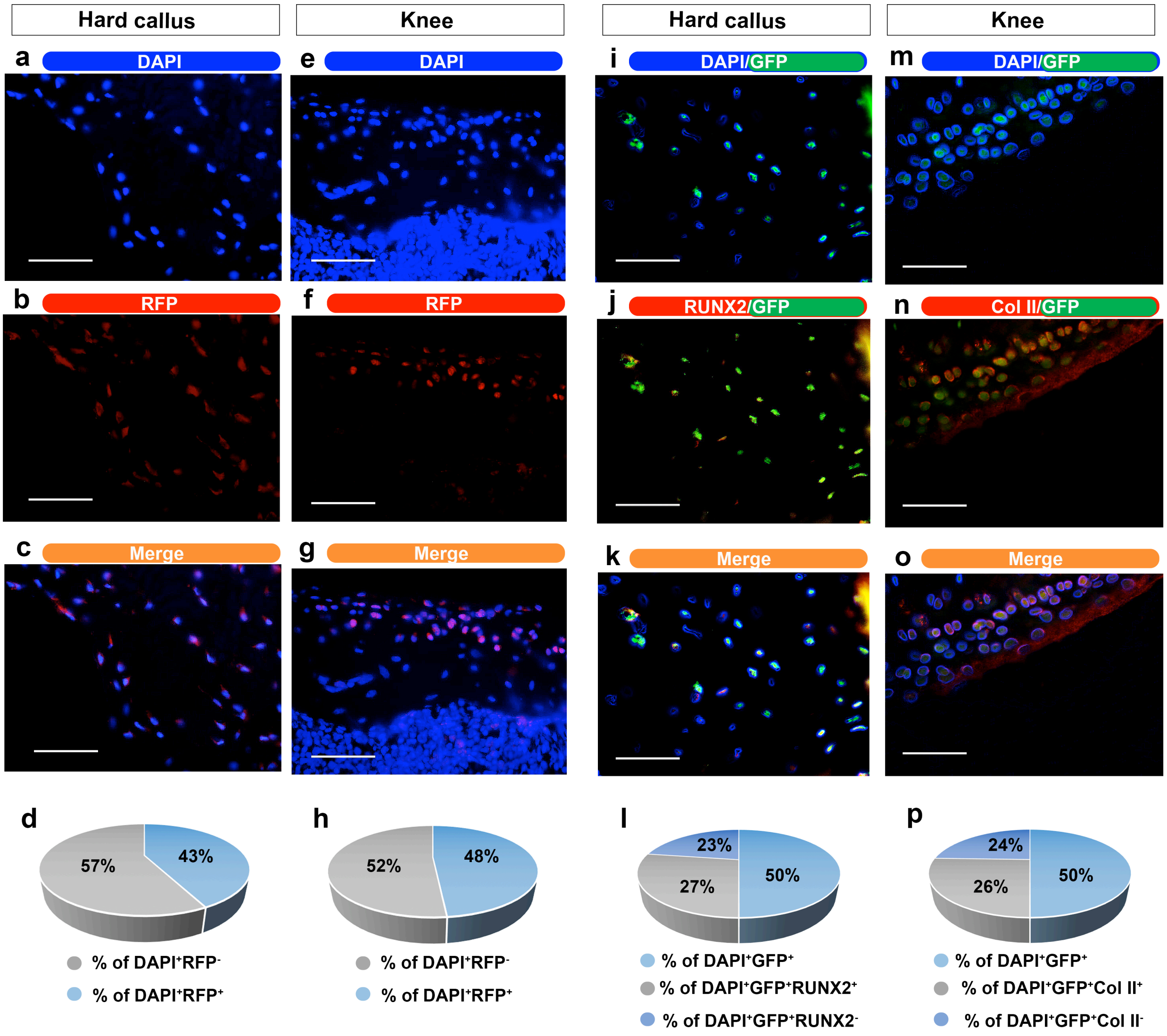

Figure S3. Quantification of BST2^{pos} CH cells migrated toward the injury sites.

(**a-g**) Representative fluorescence images used for counting RFP^{pos} and RFP^{neg} cells present in the hard callus (**a-c**) and in the knee region (**e-g**) of fractured and cell-injected mice. DAPI (blue), RFP (red). Magnification 40X; scale bar, 50 µm. (**d, h**) Quantification of total DAPI^{+}RFP^{+} and

DAPI^{+}RFP^{-} cells in the hard callus (**d**) and knee region (**h**). (**i-k**) Representative fluorescence images used for counting injected BST^{pos} CH cells present in the hard callus of fractured mice co-expressing the early osteogenic transcription factor Runx2. DAPI (blue), GFP (green), RUNX2 (red). Magnification 40X; scale bar, 50 µm. (**l**) Quantification of DAPI^{+}GFP^{+}RUNX2^{+} and DAPI^{+}GFP^{+}RUNX2^{-} within DAPI^{+}GFP^{+} migrated cells, present in the hard callus of fractured and cell-injected mice. (**m-o**) Representative fluorescence images used for counting BST^{pos} CH cells detected in the articular cartilage of fractured and cell-injected mice co-expressing type II Collagen (Col II). DAPI (blue), GFP (green), Col II (red). Magnification 40X; scale bar, 50 µm. (**p**) Quantification of DAPI^{+}GFP^{+}Col II^{+} and DAPI^{+}GFP^{+}Col II^{-} within DAPI^{+}GFP^{+} migrated cells, present in the knee region of fractured and cell-injected mice.

Figure S4

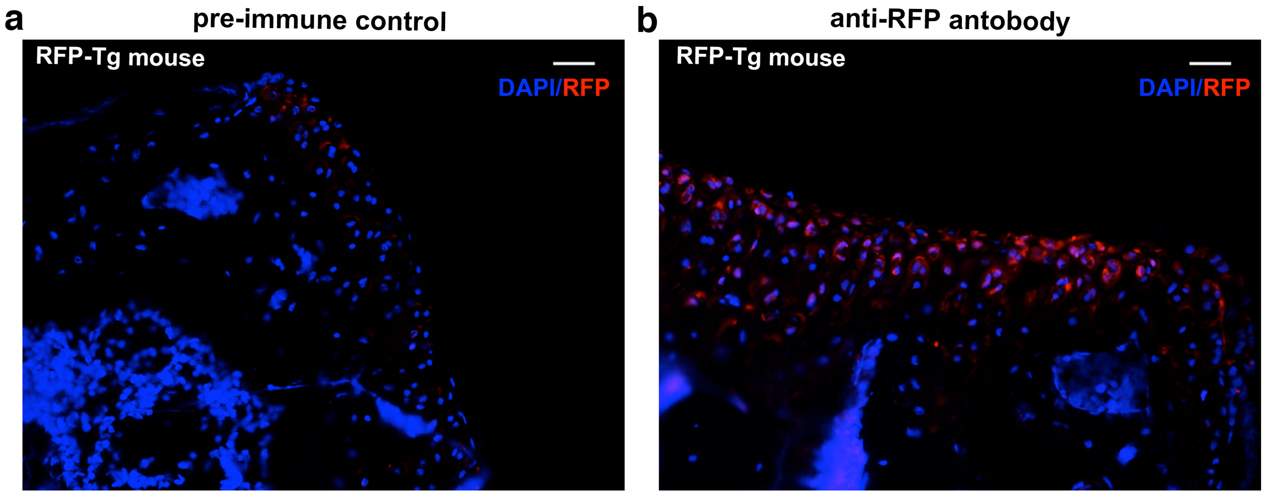

Figure S4. Specificity of the used anti-RFP antibody.

Representative fluorescence analysis of bone tissue derived from RFP-transgenic mice conducted using a specific anti-RFP antibody. (**a**) Negative control with pre-immune serum; (**b**) Positive control with anti-RFP antibody. Each panel shown the overlap between DAPI (blue) and RFP (red)

signal. Magnification 20X; scale bar, 50 µm.

Figure S5

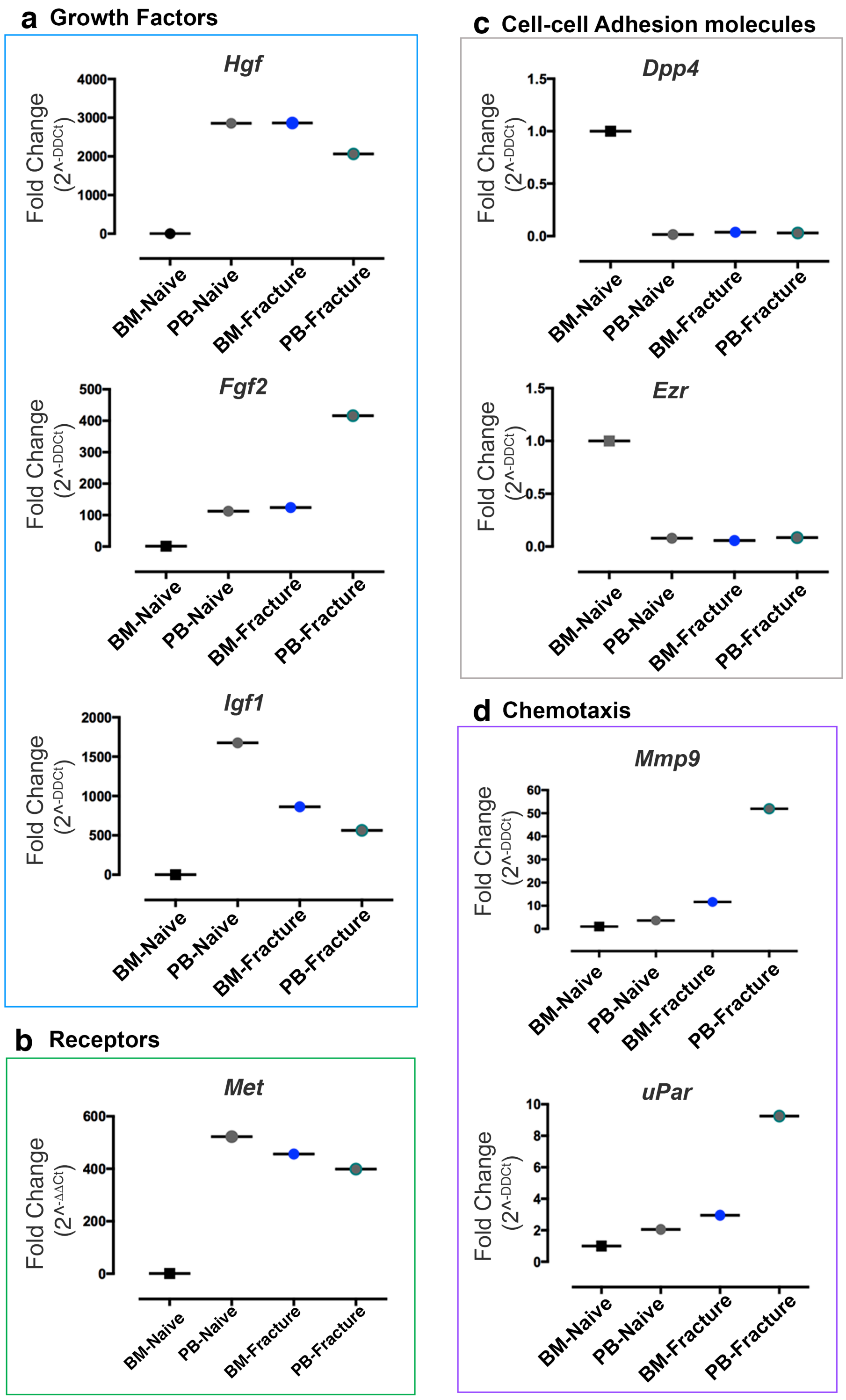

Figure S5. Effects of injury-related signals on $BST2^{pos}$ CH cells motility. Each graph reports the fold change distribution, derived from RT^2-PCR array analysis, of growth factors (**a**), receptors (**b**),

cell-cell adhesion molecules (**c**) and chemotaxis (**d**) selected protein coding genes, observed in BST2^{pos} CH cells derived from PB and Bone marrow (BM) of naïve and fractured mice. BST2^{pos} CH cells derived from BM naïve are used as reference group. *Hgf* (Hepatocyte growth factor). *Fgf2* (Fibroblast Growth Factor 2). *Igf1* (Insulin Like Growth Factor 1). *Met* (Hepatocyte growth factor receptor). *Dpp4* (Dipeptidylpeptidase 4). *Ezr* (Ezrin). *Mmp9* (Matrix metallopeptidase 9). *uPar* (Plasminogen activator or Plaur).
